# Prevalence of hepatitis-B virus co-infection among people living with HIV in Mthatha region of South Africa

**DOI:** 10.4314/ahs.v23i1.17

**Published:** 2023-03

**Authors:** Ramprakash Kaswa, Marietjie de Villiers

**Affiliations:** 1 Department of Family Medicine and Rural Health, Walter Sisulu University, South Africa; 2 Department of Family Medicine and Primary Care, Stellenbosch University, South Africa

**Keywords:** HBV, HIV, PLWH, UTT, morbidity, mortality

## Abstract

**Background:**

Hepatitis-B virus (HBV) co-infection among people living with HIV (PLWH) is highly endemic in South Africa. Despite the availability of an effective vaccine for the last four decades, chronic HBV infection is a major cause of morbidity and mortality among PLWH. Although the incidence of most opportunistic infections has been reduced in individuals with HIV since the implementation of the universal test and treat program in South Africa, HBV co-infection among PLWH is still accounting for high morbidity and mortality.

**Methodology:**

This cross-sectional descriptive survey was conducted in King Sabata Dalindyebo sub-district municipality in the Eastern Cape Province of South Africa to determine the prevalence of HBV co-infection among PLWH.

**Results:**

Two-thirds (65.5%) of the 602 PLWH who participated in the study had been screened for HBV co-infection. The mean age of the participants was 38.8±10.5 years and the majority (75.1%) were female. The prevalence of HBV co-infection among PLWH was 12.2%; among males were three times more frequently than females (OR=3, 95% CI 1.6-5.6, p=0.001). The median CD4 count of participants was 508 cell/mm^3^ (inter-quadrantile range = 307 to 715) and there was no significant association between HBV co-infection and CD4 count.

**Conclusion:**

There is a high prevalence of HBV co-infection among PLWH in the Mthatha region of South Africa. The high prevalence of HBV co-infection indicates the need for routine screening for hepatitis B among PLWH in South Africa.

## Introduction

Globally an estimated 10% of people infected with the human immunodeficiency virus (HIV) have chronic hepatitis B virus (HBV) co-infection. Around one-third of those chronically infected die as a result of serious liver disease^1^,^2^. According to the WHO global hepatitis report, HBV infection is responsible for an estimated 820 000 deaths every year^2^. An estimated 2.6 million PLWH have HBV co-infection in Sub-Saharan Africa^3^. A recent systemic review by Bigna et al (2018) reported the prevalence of HBV co-infection among people living with HIV (PLWH) as ranging from 6% to 20%, varying in the different geographic regions of Africa^4^. The Western Pacific and African regions together accounted for two-thirds (68%) of the global burden of HBV^2^,^3^. A range of global prevalence (5.1% to 7.6%) was reported, depending on gender, ethnicity, and geographic areas^2^,^4^.

The HIV epidemic is accountable for higher risk of HBV transmission and HIV-infected people are at about six times higher risk of developing chronic HBV infection than their HIV-negative counterparts^4^. Co-infection also promotes an aggressive disease course by increasing HBV replication and reactivation leading to acute liver failure, progression to liver fibrosis, and cirrhosis. Patients with chronic HBV infection are predisposed to hepatocellular carcinoma occurring at a younger age and higher risk of antiretroviral therapy (ART) related hepatotoxicity^5^. In the absence of ART, about 40% of the hepatitis B surface antigen- (HBsAg) positive patients develop progressive liver disease, cirrhosis, hepatocellular carcinoma, and terminal liver failure^6^.

HBV co-infection among PLWH remains a major health concern in sub-Saharan Africa, including South Africa^6^. Both diseases have the same route of transmission and are endemic in sub-Saharan Africa^7^. There has been recent clinical interest in HBV co-infection among PLWH owing to its chronicity. Furthermore, there is evidence that HBV/HIV co-infection is associated with higher morbidity and mortality than the individual infection on its own^7^. Although the recent success of ART has dramatically decreased opportunistic infection and increased the survival rate among PLWH, the previously unrecognized chronic HBV infection in these groups resurfaced. Importantly, first-line ART can mask undiagnosed HBV co-infection, but there is a threat of a hepatitis flare-up during a change in the ART regimen^8^.

Despite the availability of safe and effective vaccines over the last three decades, chronic HBV infection is a major cause of morbidity and mortality. The vaccine has been freely available in South Africa as part of the childhood expanded program on immunizations (EPI) since April 1995 9. The burden of chronic hepatitis B infection among PLWH in South Africa is mostly underestimated. HBV co-infection among PLWH carries a higher risk of HBV infectivity and reactivation due to the alteration of immunity by HIV infection 10. This is further complicated by the lack of routine screening and surveillance, especially in primary health care (PHC) settings. Although several studies have evaluated the prevalence of HBV co-infection among PLWHA in South Africa, there is a paucity of HBV/HIV co-infection data in rural PHC settings. This study aimed to evaluate the studies on the prevalence of HBV co-infection among PLWH attending PHC settings in the Mthatha region of South Africa. This study is part of a larger project with the overarching aim of evaluating the co-morbidity of HIV and substance use, and the response of PHC services to such patients in the Mthatha region of the Eastern Cape.

## Methods

This cross-sectional descriptive survey of HBV co-infection among PLWH is part of the baseline data of the prospective cohort study, which evaluates the effect of substance use on adherence to ART and co-morbidity among PLWH who use PHC services in Mthatha. The study was conducted at the HIV Outpatient Clinic at Ngangelizwe and Mbekweni Community Health Centre at King Sabata Dalindyebo (KSD) sub-district municipality in the Eastern Cape Province of South Africa. Mthatha is the main town in the KSD sub-district municipality. IsiXhosa is the dominant language of communication and most people depend on social welfare grants and state facilities for health care services.

### Selection of patients

**Figure 1:**
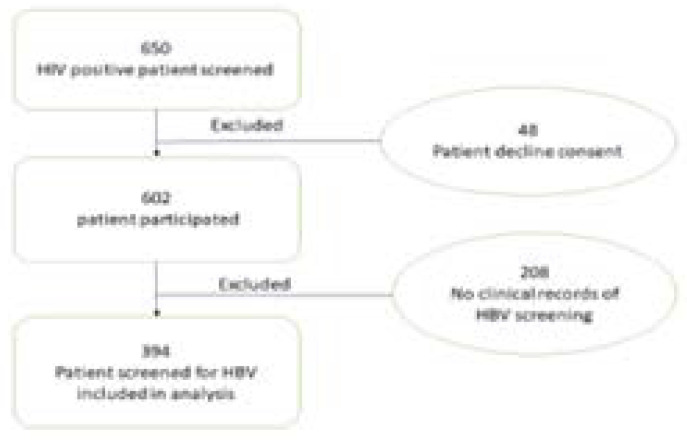
Patients flow from screening to enrolment for HBV co-infection among PLWH.

The recruitment goal of the cohort study was to enrol 600 patients who visited the HIV clinic (between 15 June to 15 August 2018); they were invited to participate in this study. Eligible subjects included HIV-infected adults (≥18 years) who had been on ART for more than six months. Patients who were severely ill or refused consent to participate were not included in the study. A flow chart of study participants is demonstrated in Figure 1. The study was approved by the Health Research Ethics Committee (HREC) of Stellenbosch University (HREC reference number: S18/01/001). The study was also approved by the Department of Health, Eastern Cape (NHRD reference number EC_201803_007), as well as the local health authorities.

### Clinical and laboratory measurement of participants

A structured questionnaire was administered by trained research assistants. The questionnaire included questions on age, sex, history of present or past self-described smoking, alcohol, and other substance use. National Health Laboratory-based fully automated chemiluminescent micro particle immunoassay (Abbott Architect HBsAg QT) was used for the detection and quantitation of hepatitis B surface antigen (HBsAg).CD4 count, and viral load results from the last 12 months were retrieved from clinical records.

### Data analysis

Descriptive statistical analysis was performed using SPSS version 18. Categorical data were reported as frequency and percentages and scale data were reported as mean and standard deviation or median and interquartile ranges if not normally distributed. Differences in demographic and clinical characteristics between patients with and without HBV infection were assessed using the chi-squared or Fisher's exact test for categorical variables and p-value <0.05 considered at significance level.

## Results

Six hundred and two HIV-infected patients participated in the baseline cohort study. After 208 (32.4%) patients had been excluded because no clinical records of HBV screening were available, the remaining 394 (65.6%) were analysed for the prevalence of HBV co-infection. The mean age of the participants was 37.8 years (SD±10.5). Most participants were female (75.1%), unemployed (70.8%), and had an education level of secondary school (85.2%). Majority of patient were on first line TEE (Tenofovir + Emtricitabine + Efavirenz) ART regimen, except four patients were on protease based second line ART regimen. The prevalence of HBV co-infection among PLWH was 12.2% and males had a three times higher infection rate than females, with a statistically significant difference (OR=3, 95%CI 1.6-5.6, p=0.001). Most HBV co-infection among PLWH occurred in the age group between 26 and 45 years. People younger than 36 years had the lower proportion of HBV co-infection compare to older counterpart, but the difference was not statistically significant (OR=0.85 95%CI 0.46-1.56 P=0.8). Substance use (Tobacco, alcohol and cannabis) were reported among PLWH but there was no significant association between HBV co-infection and substance use. The median CD4 count of participants was 508 cell/mm3, with an inter-quadrantile range of 307 to715. The prevalence of HBV co-infection was marginally higher among PLWH who had an HIV viral load of ≥ 1000 copies/ml. Table 1 represents the demographic and clinical characteristics of a lifetime and current substance use among PLWH.

**Table 1:** Demographic characteristics of hepatitis-B co-infection among people living with HIV (n=394)

	Hepatitis-B		Odd Ratio	95% confidence interval	P-value
	Positive	Negative			
**Age (Years)**					
18–35	20 (11.2%)	158 (88.8%)	0.85	0.46 – 1.56	0.8
36 and above	28 (12.9%)	188 (87.1%)			
**Gender**					
Male	22 (22.4%)	76 (77.6%)	3.0	1.6 – 5.6	0.001*
Female	26 (8.8%)	270 (91.2%)			
**CD4** count (cell/mm^3^)					
< 200	6 (14.3%)	36 (85.7%)	1.3	0.53 – 3.45	0.52
= 200	34 (10.9%)	277 (89.1%)			
**Viral load** (copies/ml)					
< 1000	32 (10.7%)	268 (89.3%)	0.53	0.2 – 1.4	0.19
=	1000	6 (18.2%)	27 (81.8%)		
**Rapid plasma regain (RPR) test**					
Positive	3 (14.3%)	18 (85.7%)	1.4	0.38 – 5.31	0.5
Negative	18 (10.5%)	154 (89.5%)			
**Alcohol use**					
Yes	28 (14.2%)	169 (85.8%)	0.68	0.37 – 1.26	0.2
No	20 (10.2%)	176 (89.8%)			
**Tobacco use**					
Yes	20 (17.4%)	95 (82.6%)	0.53	0.28 -0.98	0.04*
No	28 (10%)	251 (90%)			
**Cannabis use**					
Yes	6 (27.3%)	16 (72.7%)	0.34	0.12 -0.91	0.02*
No	42 (11.3%)	329 (88.7%)			

* P<0.05

**Figure 2:**
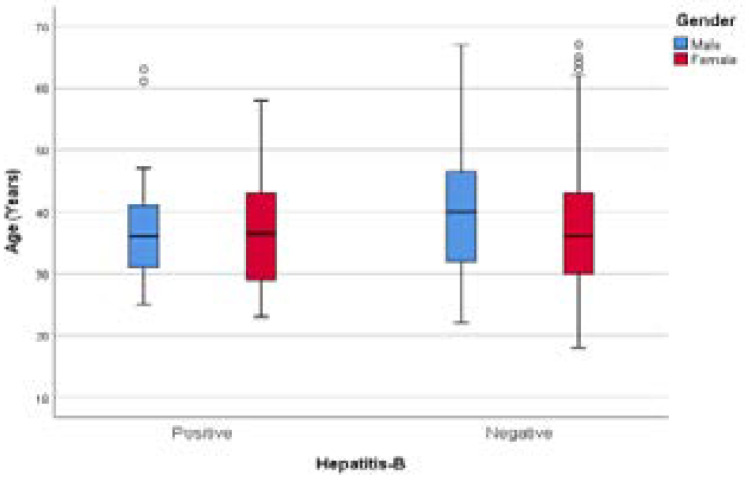
Age distribution of hepatitis-B co-infection among PLWH.

**Figure 3:**
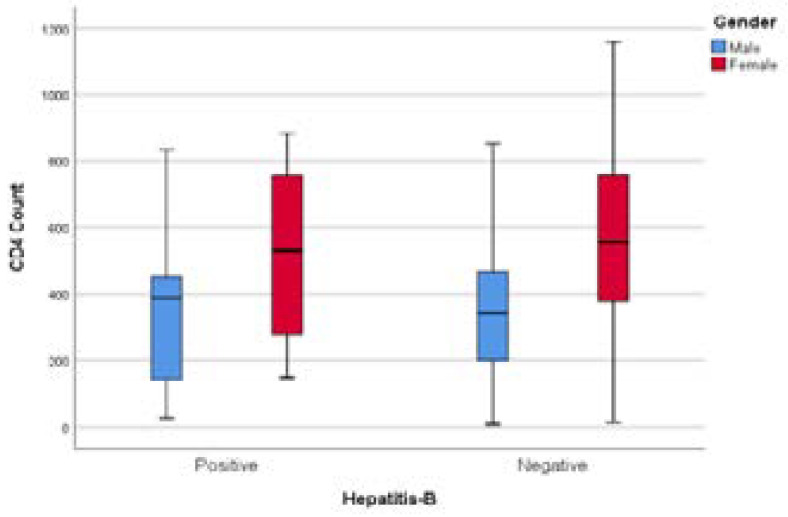
CD4 count and gender distribution of hepatitis-B co-infection among PLWH.

The box an d whisker plot in Figure 2 demonstrated that young males were more likely to be co-infected with HBV than their older counterparts. More outliers were noticed in both genders in hepatitis B-negative groups. Figure 3 demonstrated that male participants had a lower median CD4 count than their female counterparts, but there was no difference in CD4 count and HBV infection in both genders.

## Discussion

The study reported that HBV co-infection is common among PLWH. The prevalence of HBV co-infection among PLWH was similar to that found in a systemic review and meta-analysis by Bigna et al (2018) 4. They reported that overall, 12.9% of PLWH in Africa have HBV co-infection; these findings are similar (12.2%) to those of this study 4. The prevalence of HBV co-infection among PLWH was reported in a range of 6% to 20% in the different geographic regions of Africa 11, 12. Some studies from South Africa also reported a higher prevalence of HBV co-infection. A study conducted by Chonco and Rangiah reported a much higher (25%) prevalence of HBV co-infection among PLWH in Durban 7. Another study from Limpopo reported about 20% PLWH to have HBV co-infection 11. These findings can be explained by endemic distributions of HBV infection in different geographic regions.

Despite the World Health Organization's recommendation of baseline screening of HBV in a country where the prevalence is higher than 2%, about one-third (32.4%) of the participants had not been screened for HBV co-infection. Recently American and European guidelines also recommended routine screening of HBV infection among newly diagnosed HIV patients 2. The universal test and treat (UTT) program of South Africa also recommended baseline screening for HBV among all HIV-infected people. Despite the endemic nature of HBV infection in South Africa, routine screening for HBV among PLWH is still not implemented 13. The current study findings support these phenomena.

There is a large reservoir of chronic HBV infection in sub-Saharan Africa^1^. Identification is very difficult because chronic HBV infection is asymptomatic and only presents when complications arise. To prevent the life-threatening complications of HBV co-infection among PLWH, such as cirrhosis, liver failure, and hepatocellular carcinoma, early identification of individuals who are infected is essential^5^,^14^. An integrated screening program is needed to determine the real impact of HBV on the HIV epidemic in South Africa.

A significant gender difference (p = 0.001) in the prevalence of HBV co-infection among PLWH was demonstrated in the current study. The HBV co-infection was three times higher among males than among females. Most studies conducted in South Africa demonstrated a similar trend of male-predominance of HV/HIV co-infection^11^, ^14^. Various studies have shown that men are more likely to become chronic carriers of the HBV than women. The presence of the virus' surface antigen and DNA virus titers in their blood is also higher in men than women. It has been theorized that the higher rates of substance use among male patients could explain the link between these two factors^3^,^12^,^17^.

Androgens can also affect the development of the HBV through their direct binding to the Androgen Response Elements (AR) in the enhancer of the virus. This effect generates increased viral titers in serum of male subjects. Also, the presence of estrogen can affect the host's response. For instance, the natural estrogen known as 17-beta estradiol can reduce the synthesis of the cytokine Interleukin-6 by Kupffer cells^3^,^17^. The difference in the response of male and female patients to the vaccine has been suggested as a potential indicator of the lack of immunological response to treatment^14^,^16^. Multiple sexual partners are more common among male than the female counterpart and the sexual root of transmission could be a possible explanation of the high prevalence of HBV co-infection among male participants^12^,^15^.

Importantly, we found that the mean age of HBV co-infected among PLWH was 37.8 years and about 90% of them were above 25 years of age. People born before 1995 (older than 25 years) had a high prevalence of HBV co-infection and this age distribution may reflect the protective effect of childhood HBV vaccination. Since 1995 the hepatitis-B vaccine has been part of the EPI in South Africa^9^.

It is well documented that the natural history of HBV infection can be influenced by the presence of HIV and the severity of immunosuppression^2^,^14^. The immunity of HIV infection is determined by CD4 count and viral load. A low CD4 count and high viral load reflect immunosuppression. Most studies in South Africa reported an association between HBV co-infection and CD4 less than 200 cells/mm^3^. The current study findings are in alignment, with a slightly higher prevalence of HV co-infection among PLWH who presented with a CD4 count of fewer than 200 cells/mm^3^ and high viral load (≥1000 copies/ml)^14^,^16^.

Furthermore, the study stated that substance (alcohol, tobacco, and cannabis) use was reported among PLWH but there was no significant association between HBV co-infection and substance use. These findings contradicted the previous studies who reported the association between substance use HBV co-infection in this group of the population^3^,^6^,^11^,^15^.

## Limitation of the study

The researchers acknowledge several limitations. About one-third of patients were excluded because no clinical records of HBV screening were available. Patients presenting with severe illness were excluded from the study; the prevalence of HBV co-infection among this group could be different. Furthermore, the sample was derived from a selected population of PLWH who were PHC users in a local context.

## Conclusion

There was a high prevalence of HBV co-infection among PLWH attending PHC settings in the Mthatha region of South Africa. Despite the high burden of HBV co-infection, routine screening of HBV among PLWH in PHC settings was not done. Therefore, a comprehensive and integrated HBV screening program is recommended for adequate management and follow-up of hepatitis B infection among PLWH. Primary prevention through vaccination is showing results by decreasing HBV co-infection among people born after 1995. The policymaker is advised to scale up vaccination coverage to interrupt the transmission of HBV infection among PLWH.
